# Regulation of the Autophagic Bcl-2/Beclin 1 Interaction

**DOI:** 10.3390/cells1030284

**Published:** 2012-07-06

**Authors:** Jean-Paul Decuypere, Jan B. Parys, Geert Bultynck

**Affiliations:** Laboratory of Molecular and Cellular Signaling, Department of Cellular and Molecular Medicine, KU Leuven, O/N-1, bus 802, Herestraat 49, Leuven, BE-3000, Belgium; Email: jan.parys@med.kuleuven.be

**Keywords:** autophagy, Bcl-2, Bcl-X_L_, Mcl-1, Beclin 1, JNK1, DAPK, IP_3_R, Naf-1, BH3 mimetics

## Abstract

Autophagy is an intracellular degradation process responsible for the delivery of cellular material to the lysosomes. One of the key mechanisms for control of autophagy is the modulation of the interaction between the autophagic protein Beclin 1 and the members of the anti-apoptotic Bcl-2 family (e.g., Bcl-2, Bcl-X_L_ and Mcl-1). This binding is regulated by a variety of proteins and compounds that are able to enhance or inhibit the Bcl-2/Beclin 1 interaction in order to repress or activate autophagy, respectively. In this review we will focus on this interaction and discuss its characteristics, relevance and regulation.

## 1. Introduction

Autophagy is an evolutionary conserved intracellular pathway responsible for the delivery of cellular material to lysosomes for its degradation. Macroautophagy (further referred to as ‘autophagy’) is the best studied form of autophagy, in part because of its unique features: the double-membranous vesicles, termed autophagosomes, which carry the cellular material towards the lysosomes. These autophagic vesicles derive from double membranous structures (phagophores) that elongate, envelop the cellular material, and finally close up to form an autophagosome [1]. The enclosed material usually consists of long-lived proteins, but also entire organelles (e.g., mitochondria, then termed mitophagy) and even foreign pathogens (xenophagy) can be transported along this pathway for degradation. The autophagosomes fuse with endosomes to form amphisomes, and finally with lysosomes to form autolysosomes. After the lysosomal degradation, the cellular material is recycled into its building blocks and transported back into the cytoplasm for further use.

Autophagy is an essential player in cellular life and death. Basal levels of autophagy control the cellular homeostasis by removing possible intrinsic toxic components, such as damaged organelles or protein aggregates. In this regard, (dysfunctional) autophagy is often implicated in various diseases (e.g., proteinopathies) [2,3]. In addition, the autophagic process becomes upregulated during various types of stress, serving as a survival mechanism. It clears cellular entities that have been damaged by the stress, while the recycling of its own material provides fresh nutrients, independently of the extracellular environment. This enables the cell to survive even in nutrient-limiting conditions. Therefore, blocking autophagy during stress normally enhances cell death. However, several studies also observed that inhibition of autophagy by phosphatidylinositol 3-kinase (PtdIns-3K) inhibitors (see below) or knock-down of autophagy proteins was protective during certain stress conditions, which led researchers to believe that too severe autophagy levels can result in cell death, termed ‘programmed cell death type II’ or ‘autophagic cell death’. Whether this really represents a cell death mechanism *by* autophagy or cell death associated *with* autophagy (e.g., where the autophagic machinery is important for the progress of other cell death mechanisms, like apoptosis or necrosis) is still a matter of debate [4,5] and should be clearly assessed in studies considering this topic. 

Another interesting controversial matter is the origin of the lipids of the phagophore. Several organelles have already been assigned as candidates, including the endoplasmic reticulum (ER) [6,7], mitochondria [8], the Golgi apparatus [9] and the plasma membrane [10]. The ER as the origin has the most compelling evidence, including from 3D electron tomography [6,7], revealing phagophore structures arising from a specialized region in the ER membrane (termed ‘omegasome’) in a very specific way. However, this does not exclude the other candidate membranes as possible lipid sources for the phagophore, since they could contribute lipids for the further elongation. Moreover, the employed lipid source may depend on the specific type of stress, or of the damage to a specific organelle.

## 2. The Phosphatidylinositol 3-Kinase Complex III

In yeast, more than 30 autophagy (‘Atg’) proteins have already been reported that molecularly regulate the autophagic pathway. This pathway partially overlaps with the vacuolar protein-sorting (‘Vps’) pathway. This is especially apparent in one of the complexes essential for the initial formation of the phagophore, the PtdIns-3K complex III (or PtdIns-3KC3), of which the key kinase is named Vps34 [11]. The complex further consists of Vps15 and Vps30/Atg6. Whether this complex functions in the autophagic pathway or in the Vps pathway depends on the other components of this complex (see below). Active Vps34 will phosphorylate phosphatidylinositol (PtdIns) to form phosphatidylinositol-3-phosphate (PtdIns3P) in the membrane platform for the formation of autophagosomes, thus creating PtdIns3P-rich omegasomes. This PtdIns3P serves as a recruitment signal for other autophagy proteins, such as Atg18 (WIPI-1 and WIPI-2 in mammals) and Atg21, which then positively regulate the further elongation of the phagophore [12,13,14]. Note that Vps34 and its product PtdIns3P also activates the mammalian target of rapamycin mTOR downstream of amino-acid presence [15,16], through a mechanism that involves the recruitment and activation of phospholipase D1 by PtdIns3P at the lysosomes [17]. This would imply an inhibition of autophagy, but these differences are likely to reflect two distinctly localized complexes [18].

The mammalian ortholog of the essential PtdIns-3KC3 component Vps30/Atg6, Beclin 1, has gained a special interest in autophagy research, because it was the first autophagy protein shown to be a haploinsufficient tumor suppressor (Table 1) [19,20]. This discovery has further boosted the entire autophagy research field since then [21]. Beclin 1 interacts with its C-terminal evolutionary conserved domain (ECD, a.a. 244–337) [22] together with its coiled-coil domain (CCD, a.a. 150–244) [23] to the N-terminal C2 domain of Vps34 (a.a. 1–250), and this interaction is required for Vps34 activity. Since Beclin 1 also contains a BH3 domain (a.a. 114–123), it is a member of the apoptotic BH3-only protein family [24]. However, while a peptide corresponding to the BH3 domain of Beclin 1-induced apoptosis [25], full-length Beclin 1 did not [26], probably because the other surrounding domains suppress the pro-apoptotic characteristics of its BH3 domain. Furthermore, a recent study clearly showed that Beclin 1 is not implicated in apoptosis [27]. Beclin 1 functions as a platform molecule for the PtdIns-3KC3, binding several proteins via its CCD or BH3 domain, thereby fine-tuning the activity of Vps34.

**Table 1:** List of examples of phenotypes observed in mice deficient in Beclin 1, Bcl-2, Bcl-X_L_, Mcl-1 and Bcl-w. When homozygous (−/−) knockout mice are embryonically lethal, heterozygous (+/−) or conditional knockout (CKO) mice phenotypes are also listed.

Mice	Phenotypes	References
Beclin 1-/-	Prenatal embryonic lethality	[19,20]
Beclin 1+/-	Increased tumor incidence	[19,20]
	Abnormal ovary morphology	[38]
	Increased cell proliferation	[19,39]
	Premature death	[19]
	Increased angiogenesis	[39]
	Decreased cardiac injury	[40,41]
	Neurodegeneration	[42]
	Renal fibrosis	[43]
Bcl-2-/-	Partial postnatal lethality	[44,45,46,47,48,49,50]
	Abnormal kidneys/Polycystic kidney disease	[44,45,46,47,48,49,50,51]
	Abnormal lymphoid system	[44,45,46,48,50]
	Abnormal spleen morphology	[46,48,50,52]
	Abnormal thymus morphology	[48,50]
	Small ears	[44,45,48,50]
	Abnormal nose morphology	[44,48]
	Decreased body size/weight	[44,45,46,47,48,50]
	Abnormal hair pigmentation	[44,45,48,50]
	Abnormal neuron morphology/Neuron degeneration	[47,53]
	Abnormal retinal vasculature morphology/Pericyte morphology	[54]
	Abnormal skeletal muscle fiber type ratio	[55]
	Abnormal small intestine morphology	[50]
	Abnormal osteoblast morphology	[56]
Bcl-XL-/-	Prenatal lethality	[57,58,59]
Bcl-XL-/- (CKO)	Abnormal platelet morphology/physiology	[57]
	Neuron degeneration	[58,59]
	Liver fibrosis	[60]
	Anemia/Splenomegaly	[61]
	Increased bone resorption	[62]
Mcl-1-/-	Peri-implantation embryonic lethality	[63]
Mcl-1-/- (CKO)	Decreased T cell number/Abnormal T cell morphology	[64,65]
	Decreased neutrophil number	[66]
	Decreased mast cell and basophil number	[67]
	Decreased thymocyte number	[68]
	Neuron degeneration	[69]
	Abnormal liver morphology	[70,71,72]
Bcl-w-/-	Male infertility	[73,74,75]
	Abnormal neurological behavior	[76]
	Decreased cellular stress protection	[77,78]

Although Beclin 1 is regarded as essential for Vps34 activity and thus for the autophagic pathway, some reports indicate the existence of non-canonical Beclin 1-independent autophagy. Interestingly, in these studies, the Beclin 1-independency mostly resulted in autophagic cell death, in which inhibition of autophagy leads to more survival [28,29,30]. This would suggest that Beclin 1 levels might contribute to the decision whether autophagy will be protective or detrimental [26]. However, since Beclin 1-dependent autophagic cell death has also been described (e.g., [31,32,33]), other factors will also play a role in the autophagic decision to counteract or amplify cell death.

## 3. The Anti-Autophagic Bcl-2-Family Proteins

Beclin 1 was first discovered as a Bcl-2-interacting protein important in protection against Sindbis virus [34]. Further investigation by the same research group revealed that this interaction was modulated during autophagy stimulation. In normal conditions, Bcl-2 inhibits Beclin 1, while upon stress Beclin 1 dissociates from Bcl-2, allowing the activation of Vps34 and the subsequent stimulation of autophagy [26]. Since the BH3 domain of Beclin 1 is important for Bcl-2 binding and hence autophagy inhibition, it is not surprising that Beclin 1 also interacted with other Bcl-2 family members: Bcl-X_L_, Bcl-w and Mcl-1 [24] and viral Bcl-2 proteins (see below). Typically for BH3-containing proteins, the BH3 domain of Beclin 1 binds to the hydrophobic groove in Bcl-2 proteins, formed by their BH1, BH2 and BH3 domain [25,35]. The fact that Beclin 1 binds several members of the Bcl-2 family proteins implies that the regulation and the physiological importance and role of this interaction will differ depending on its binding partner, which is also reflected in the differences among the phenotypes of the respective knockout mice (Table 1). Since the Bcl-2/Beclin 1 interaction seems to function as a key regulatory mechanism in autophagy, a plethora of proteins and compounds have been described that alter autophagy through regulation of this interaction (see below).

Since Bcl-2 is very well known as an anti-apoptotic protein, the mechanism by which Bcl-2 inhibits both a survival as well as a cell death process is intriguing. In this respect, Wei *et al.* [36] reported that a short period of nutrient deprivation (4 h) led to the dissociation of Beclin 1 from Bcl-2, but not of Bax. Longer periods (16 h) however also led to dissociated Bax. This suggests that subtle regulations of Bcl-2 can already release Beclin 1, and therefore activate autophagy, while more severe alterations in Bcl-2 are necessary to dissociate pro-apoptotic proteins and stimulate apoptosis. In this respect, the binding of Beclin 1 to Bcl-2 proteins has been shown to be weaker than for pro-apoptotic BH3 proteins [25,37].

**Figure 1:**
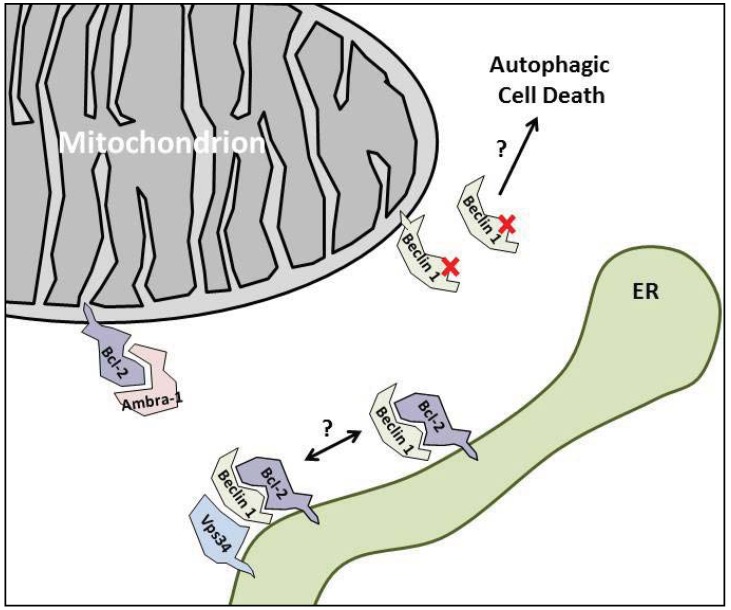
Bcl-2 tethers Beclin 1 at the ER. Representation of mitochondria, ER and the proteins of interest in normal growth conditions. Since only ER-Bcl-2 regulates autophagy, it is able to recruit Beclin 1 at the ER. Bcl-2-binding mutants of Beclin 1 (indicated with a red cross), therefore are less able to interact with Vps34 at the ER and fail to induce autophagy or could induce autophagic cell death. Whether Bcl-2/Beclin 1 complexes are devoid of Vps34 is still unclear. Beclin 1 can also associate with mitochondria. Ambra-1 binds mitochondrial Bcl-2 and prevents it from binding to Beclin 1. See text for detailed information.

An interesting aspect of Bcl-2 is that its anti-autophagic function appears to be dependent on the intracellular localization. Wild type and ER-localized Bcl-2 are able to repress autophagy, while mitochondrial Bcl-2 is not [26]. It should be noted however, that the possibility to inhibit autophagy also depends on the stress, as ER-targeted Bcl-2 and Bcl-X_L_ were able to inhibit starvation-induced, but not ER stress-induced autophagy [79]. An important question is why Bcl-2 at the mitochondria does not regulate autophagy. A possible explanation is that at the ER, Bcl-2 can also inhibit autophagy by lowering the ER Ca^2+^ content [80], although this does not involve Beclin 1. Another possibility is that proteins regulating this interaction are mainly present at the ER membranes (see below). In line with this view, it was shown that Beclin 1 still interacted with mitochondrial Bcl-2, but did not dissociate upon starvation or treatment with the BH3-mimetic molecule ABT-737, in contrast to wild type or ER-localized Bcl-2 [25]. Finally, we could argue that in an unbound state, autophagic Beclin 1 interacts with Vps34 at the omegasomes on the ER [81]. Thus, efficient regulation of Beclin 1 by associated proteins like Bcl-2 likely requires their tethering to ER membranes. *Vice versa*, this would mean that Beclin 1 bound via Bcl-2 at the ER is also tethered in the best position to interact with Vps34, once it becomes dissociated (Figure 1). In this manner, Bcl-2 also may have a subtle pro-autophagic function by recruiting Beclin 1 at the ER. This hypothesis can explain why increased autophagy was observed in etoposide-treated mouse embryonic fibroblasts overexpressing Bcl-2 or Bcl-X_L_ [82] and why overexpression of a Beclin 1 mutant lacking the Bcl-2-binding domain in hl-1 cardiac myocytes resulted in less starvation-induced autophagy [83]. However, the former did not occur in HeLa, Jurkat or HCT116 cells [82] and the latter was not observed in MCF7 cells [26] or HeLa cells [25], possibly due to cell-type dependent differences. In this respect, although overexpression of the Bcl-2-binding mutant of Beclin 1 in MCF7 cells led to increased autophagy, it was not associated with increased Vps34-Beclin 1 binding [26], suggesting another unknown mechanism for the observed increased autophagy in these cells. Interestingly, overexpression of those Beclin 1 mutants defective in Bcl-2 binding increased starvation-induced autophagic cell death in several cell types, compared to wild type Beclin 1 [26], suggesting that improper tethering of Beclin 1 at the ER results in a greater sensitivity towards autophagic cell death, via another Vps34-independent mechanism (Figure 1). Possibly, loss of recruitment of Beclin 1 at the ER will result in more mitochondrial Beclin 1, since it contains a membrane-binding domain in its C-terminal ECD that prefers mitochondrial cardiolipin-enriched membranes [84] (Figure 1). In summary, Bcl-2 prevents autophagy, but may also be required for it to function as a survival process.

Although the Bcl-2/Beclin 1 interaction is clearly an important checkpoint in autophagy induction upon starvation or treatment with many different compounds, some other well-known autophagy inducers, like rapamycin (an mTOR inhibitor) and lithium chloride (inhibitor of inositol signaling), had less apparent effects on the displacement of Beclin 1 from Bcl-X_L_ [25]. However, they induce autophagy in a Beclin 1-dependent manner. Since only Bcl-X_L_ was investigated, rapamycin and lithium may only affect another Beclin 1 pool, complexed to other proteins (e.g., Bcl-2, Mcl-1, nPIST [85]). Another explanation would be that Beclin 1, complexed to Bcl-2 proteins, may be activated without displacement from Bcl-2 upon these treatments, dependent on other unknown factors. Although in *C. elegans* there is evidence that Beclin 1, Vps34 and Bcl-2 do not form a tripartite complex [86] and Bcl-2 overexpression disrupted the interaction between Vps34 and Beclin 1 [26], other reports imply otherwise. Vps34/Beclin 1/Bcl-2 complexes were observed in mammalian cells, albeit upon overexpression of the three respective proteins [23], and ABT-737, a BH3 mimetic that decreases the Bcl-2/Beclin 1 interaction, had no effect on Vps34/Beclin 1 association [87]. These latter results suggest that such tripartite complexes can exist, but the presence of Bcl-2 would keep Beclin 1 in an inactive conformation (Figure 1). Perhaps complexed with Bcl-2, Beclin 1 may have some very minor activity, enough to induce basal levels of autophagy, but not the high levels that are reached during starvation. This is evidenced by studies using mutant JNK1, a key regulator of the Bcl-2/Beclin 1 interaction (see below), which only affects starvation-induced, but not basal autophagy [88]. Also, Bcl-2-AAA mice, which cannot be regulated by JNK1, have no differences in basal autophagy [89]. In conclusion, the inhibition of the Bcl-2/Beclin 1 interaction seems not to be a complete requisite for all types of autophagy induction, and how Beclin 1 is regulated, and which partners it binds to during autophagy upon different treatments remains to be further investigated.

## 4. Enhancing the Bcl-2/Beclin 1 Interaction

Many proteins that interact with Beclin 1 to regulate its function in autophagy, including UVRAG, Atg14L/Barkor, Rubicon, Bif-1, nPIST, SLAM have been reported (reviewed in: [90]), but whether these proteins modulate Bcl-2/Beclin 1 interaction is yet unclear. In addition, proteins that affect Beclin 1 transcription, e.g., p65/RelA [91] or E2F1 [92], can indirectly affect this interaction through regulation of Beclin 1 levels, but do not directly interact with Bcl-2 or Beclin 1. Therefore, these Beclin 1-regulating proteins are not subject of this review. Here, we will focus on those regulatory proteins shown to regulate Bcl-2/Beclin 1 interaction and to interact with one of the partners, because of the potential therapeutic applications of modulation of this binding (see below).

### 4.1. Parkin

**Figure 2:**
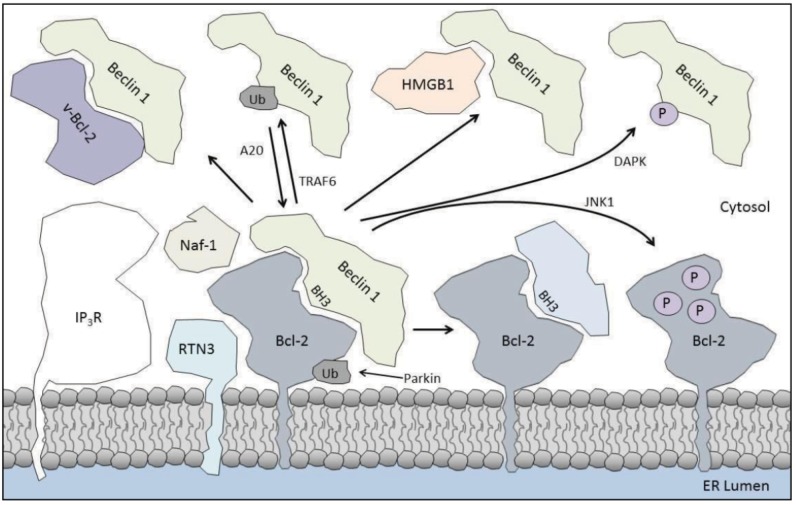
Regulation of the Bcl-2/Beclin 1 interaction at the ER. The Bcl-2/Beclin 1 interaction can be enhanced by RTN3, Naf-1, IP_3_R and ubiquitination of Bcl-2 by Parkin. Dissociation of Beclin 1 from Bcl-2 is promoted by BH3-only proteins binding to Bcl-2, phosphorylation of Bcl-2 by JNK1, phosphorylation of Beclin 1 by DAPK, viral Bcl-2 (v-Bcl-2) or HMGB1 binding to Beclin 1 or ubiquitination of Beclin 1 by TRAF6. The latter can be counteracted by deubiquitinating enzyme A20. See text for detailed information.

Mutations in the *parkin* gene cause autosomal recessive juvenile onset Parkinsonism [93]. Its major cellular function is as an E3 ubiquitin ligase, involved in protein degradation via the ubiquitin-proteasome pathway. However, Parkin also promotes mitophagy through its recruitment to dysfunctional mitochondria [94]. Apart from promoting autophagy specifically for mitochondria, Parkin also inhibits autophagy viamono-ubiquitination of Bcl-2 [95]. However, this ubiquitin signal did not increase the degradation of Bcl-2, but augmented Bcl-2 levels due to increased Bcl-2 stability. Co-immunoprecipitation studies revealed a clear interaction between Parkin and Bcl-2, but not Bcl-X_L_. The enhanced stability of Bcl-2 consequently resulted in a stronger binding between Beclin 1 and Bcl-2, which was dependent on the presence of Parkin (Figure 2). By using mutated Parkin, the E3 ubiquitin ligase activity was found to be required for these effects. Interestingly, several disease-linked mutations of Parkin that impair its ligase activity too failed to inhibit autophagy [95].

The mechanism by which Parkin induces mitophagy while inhibiting autophagy in general probably depends on its localization. Damage to mitochondria will target Parkin to the mitochondria to promote mitophagy and clearance of these affected organelles, while upon a more global stress like starvation, Parkin may be retained in the cytosol [95], where it serves an anti-autophagic function. 

### 4.2. IP_3_R

The main ER-resident Ca^2+^-release channel, the inositol 1,4,5-trisphosphate receptor (IP_3_R), has also been implicated in autophagy (reviewed in: [96]). IP_3_R inhibition by the selective chemical inhibitor xestospongin B or by siRNA knock down or by genomic ablation resulted in increased autophagy [79,97,98,99]. However, the mechanism of IP_3_R-dependent autophagy inhibition was debated, with both Ca^2+^-dependent [98,99] and Ca^2+^-independent mechanisms [79,97] proposed. In the latter line of view, Vicencio *et al.* [97] suggested a scaffolding role for the IP_3_R, binding to both Beclin 1 and Bcl-2 separately, thereby increasing their proximity and enhancing this *in se* weak anti-autophagic interaction (Figure 2). This was supported by following experimental evidences: xestospongin B decreased the Bcl-2/Beclin 1 as well as the IP_3_R/Beclin 1 interaction, while Bcl-2 knock down also decreased the interaction of Beclin 1 with the IP_3_R. Bcl-2, Bcl-X_L_ and Mcl-1 are known to interact with the IP_3_R [100,101,102], but the proposed IP_3_R interaction site for Beclin 1 was different than that for the Bcl-2 proteins [101,103]. Beclin 1 namely interacted with the region containing the IP_3_-binding core (a.a. 225–604) [97], as well as to the suppressor domain (a.a. 1–225) [104] a region very important in controlling the Ca^2+^ channel function of the IP_3_R [105]. This scaffold hypothesis is interesting, especially since IP_3_Rs have very large cytosolic domains prone to recruiting auxiliary proteins [105,106]. However, in addition to this scaffold function, xestospongin B could also induce autophagy via inhibition of constitutive IP_3_R-mediated Ca^2+^ release events fueling mitochondrial energy production [99].

In contrast to this scaffold hypothesis, our own study revealed an increased IP_3_R/Beclin 1 interaction and a decreased Bcl-2/Beclin 1 interaction during short periods of nutrient starvation. The former interaction was responsible for an IP_3_R sensitization that was important for autophagy induction [104]. Our results indicated that the increased IP_3_R/Beclin 1 interaction resulted by itself in enhanced IP_3_R-mediated Ca^2+^-release activity. Bcl-2 was shown to be necessary for this interaction in an indirect manner, possibly through recruitment of Beclin 1 at the ER, correlating with the release of Beclin 1 from the IP_3_R by Bcl-2 knock down as reported previously [97]. In conclusion, the role of IP_3_Rs in this process, as scaffold proteins or Ca^2+^-release channels, is an important issue to resolve in the future.

### 4.3. Naf-1

Nutrient-deprivation autophagy factor 1 (Naf-1) is a key protein involved in the Wolfram syndrome 2 (WSF2) neurodegenerative disorder [107]. It is predominantly expressed at the ER, where it appears to interact with Bcl-2 and enhance ER-localized Bcl-2’s anti-autophagic functions [108]. Naf-1 knock down decreased the Bcl-2/Beclin 1 interaction and enhanced starvation-induced autophagy. Remarkably, although Naf-1 knock down led to more dissociated Beclin 1 in normal growth conditions, it did not affect basal autophagy levels. While Naf-1 selectively enhanced Bcl-2/Beclin 1 interaction, it dissociated from Bcl-2 after Bik overexpression, suggesting that Naf-1 is only required for Bcl-2’s interaction with Beclin 1, not the other BH3 proteins. In this line of view, Naf-1 may be a key factor required to stabilize the relatively weak Bcl-2/Beclin 1 interaction (Figure 2). Interestingly, Naf-1 also associated with IP_3_R1, but whether this interaction was directly or indirectly via Bcl-2 was not established. In addition, Naf-1 enhanced Bcl-2’s ability to lower the ER Ca^2+^-store content [108], which may be important for the stimulation of autophagy [80]. Increased autophagy and dysregulated Ca^2+^ homeostasis was also observed in Naf-1^−/^^−^ mice [109].

### 4.4. Reticulon 3

The reticulon family is proposed to regulate a variety of cellular processes, including retrograde transport from Golgi to ER [110], and shaping the ER [111,112]. Reticulon 3 (RTN3), a protein predominantly expressed in the brain and localized in the ER, is known to be involved in Alzheimer’s disease, likely by inhibiting β-secretase enzyme 1 (BACE1), which cleaves amyloid precursor protein [113]. In addition, recent evidence emerged that RTN3 regulates autophagy induction upon cytoplasmic prion protein (CyPrP) expression [114]. In N2A neuroblastoma cells, RTN3 was found to be transcriptionally upregulated upon cyPrP expression, probably as a result of the induced ER stress. Interestingly, knock down of RTN3 enhanced autophagy induced by the cyPrP aggregates, leading to better clearance of the aggregates, and attenuation of ER stress and apoptosis. The mechanism of autophagy inhibition was proposed to happen via regulation of the Bcl-2/Beclin 1 interaction, since RTN3 overexpression strongly increased this interaction [114] (Figure 2). In this regard, RTN3 was also shown to interact with Bcl-2 [115]. However, this interaction resulted in an enhanced anti-apoptotic function of Bcl-2, which is in contrast to the anti-autophagic and therefore pro-apoptotic function of RTN3 during PrP expression [114]. In conclusion, RTN3 appears to function as an enhancer of Bcl-2’s function, whether it is anti-apoptotic or anti-autophagic, but the exact outcome will depend on the apoptotic/autophagic trigger used. 

Interestingly, RTN3 also affected Ca^2+^ homeostasis by increasing ER Ca^2+^ release and consequently depleting ER Ca^2+^ stores [116], although the exact mechanism was not clarified. In this manner, it was shown to provoke apoptosis. However, whether RTN3 also affects autophagy by increasing cytosolic [Ca^2+^] [80,96] or by regulating IP_3_Rs in complex with Beclin 1 and Bcl-2 is still an issue to be resolved.

### 4.5. Beclin 1 Self-Association

Using analytical ultracentrifugation, Noble *et al.* [117] reported the potential for Beclin 1 to dimerize, and that viral Bcl-2 and Bcl-X_L_ bound the Beclin 1 dimer, while UVRAG interacted with the monomer. More astonishingly, Beclin 1 fragments tested by gel filtration chromatographic analysis showed that the CCD fragment of Beclin 1 forms a homotetramer, while a fragment consisting of both the BH3 domain and CCD domain (a.a. 101–267) formed a homo-octamer [118]. Similarly, Adi-Harel *et al.* [119] confirmed that Beclin 1 self-interacted and that the CCD and the N-terminal domain, but not the BH3 domain, were required. Using chemical cross-linking, they identified a trimeric Beclin 1 complex. The appearance of large complexes is completely in line with its proposed function as a platform molecule. However, in contrast to the model proposed by Noble *et al.* [117], overexpression of Vps34, UVRAG or Bcl-X_L_ had no effect on Beclin 1 self-association. In addition, the stimulation of autophagy by amino-acid deprivation or rapamycin did not alter the rate of Beclin 1 self-interaction [119], challenging the physiological regulation of this self-association, its importance for Bcl-2/Beclin 1 interaction and for controlling the autophagic pathway.

## 5. Inhibiting the Bcl-2/Beclin 1 Interaction

### 5.1. Kinases

Since ER-localized Bcl-2 regulates autophagy and phosphorylated Bcl-2 localizes mainly at the ER [120], it is not surprising that the Bcl-2/Beclin 1 interaction can be regulated by kinases that phosphorylate Bcl-2. Wei *et al.* [88] showed such a role for c-Jun N-terminal kinase 1 (JNK1), but not JNK2 (Figure 2). JNK1 becomes activated upon starvation and phosphorylates Bcl-2 at three residues in its unstructured loop between the BH4 and BH3 domain (T69, S70 and S87). Viral Bcl-2, which does not contain these phosphorylatable residues, and phospho-dead mutant Bcl-2 (Bcl-2-AAA) strongly bound Beclin 1 and failed to dissociate Beclin 1 upon starvation. A phospho-mimic mutant (Bcl-2-EEE) did not interact with Beclin 1 at all, not even in nutrient-rich growth conditions. As a consequence, overexpression of Bcl-2-EEE did not inhibit starvation-induced autophagy, in contrast to wild type Bcl-2 and Bcl-2-AAA [88]. JNK1 can both regulate survival as well as apoptosis, depending on the duration of JNK1 stimulation [121]. Also, with respect to autophagy and apoptosis, the duration of starvation was the determining factor. Short periods (4 h) of starvation led to dissociation of Beclin 1 from Bcl-2, but not of Bax. Bax dissociation happened at a much slower pace (16 h of starvation), correlating with the activity of caspase 3 [36]. This is probably due to the weaker binding of Beclin 1 to Bcl-2, compared to other BH3-containing proteins [25,37].

Interestingly, Bcl-2-AAA knock-in mice were found to have disrupted exercise-induced autophagy, resulting in abolished enhancement of the glucose metabolism [89]. However, although exercise was capable of dissociating Beclin 1 from Bcl-2, it was not associated with increased JNK activity, suggesting other kinases may be involved in exercise-induced autophagy.

Unlike Bcl-2, Bcl-X_L_ is not phosphorylated by JNK1, since it does not have similar residues, suggesting that other forms of regulatory mechanisms must exist for Bcl-X_L_. Zalckvar *et al.* [122] elucidated an important role for the calcium/calmodulin-dependent serine/threonine kinase DAPK (death-associated protein kinase) in autophagy [123,124] with its ability to phosphorylate Beclin 1 at residue T119 in its BH3 domain. Importantly, this is a unique property of Beclin 1, as a hydrophobic residue is found at this position in other BH3-only proteins. This difference may underlie the weaker binding of Beclin 1 to Bcl-2 proteins [37]. DAPK interacted with Beclin 1, for which its Bcl-2-binding site was required, phosphorylated Beclin 1 mainly at T119, dissociating Beclin 1 from Bcl-X_L_ [122] and Bcl-2 [125] (Figure 2). However, the authors only analyzed DAPK-overexpressing cells, disregarding the relevance of this phosphorylation during stress conditions as starvation. In addition, it remains to be resolved whether Beclin 1 phosphorylation affects its interaction with other Beclin 1-binding proteins, like Barkor, UVRAG or the IP_3_R.

Finally, the extracellular signal-regulated kinase ERK too has been suggested to phosphorylate Bcl-2 and affect the Bcl-2/Beclin 1 binding, which is regulated by HMGB1 [126] (see below). However, an actual interaction between the kinase and Bcl-2 has not yet been established [90], suggesting that this phosphorylation may also be caused by another kinase downstream of ERK.

### 5.2. Ambra-1

Ambra-1 (activating molecule in Beclin 1-regulated autophagy) is a known Beclin 1 interactor that positively regulates PtdIns-3KC3 [127]. It becomes disconnected from the cytoskeleton upon autophagy induction and relocalizes to the ER to regulate autophagosome nucleation [128]. Strappazzon *et al.* [129] recently also revealed an interaction between mitochondrial Bcl-2 and Ambra-1, which became disrupted upon autophagy induction. The interaction of Beclin 1 with Ambra-1 occurred via Beclin 1’s BH3 domain, suggesting Ambra-1 and Bcl-2 compete for Beclin 1. Interestingly, this interaction also happened with a Bcl-2-EEE mutant, which is unable to bind Beclin 1, suggesting that the Ambra-1 induced dissociation of Beclin 1 can happen independently of the regulatory kinases. Although several aspects remain to be clarified, the findings suggest that Ambra-1 may be important in inhibiting the tethering of Beclin 1 to mitochondrial Bcl-2 (Figure 1). Subsequently, upon autophagy induction, Ambra-1 is released from the mitochondrial Bcl-2 in order to interact with Beclin 1 and further stimulate autophagy. 

### 5.3. HMGB1

High mobility group box 1 (HMGB1) is a nuclear chromatin-binding protein that can also be secreted in the extracellular environment, functioning as a signaling molecule in e.g., inflammation, cell migration and tumor metastasis [130]. During starvation or rapamycin treatment, HMGB1 translocates from nucleus to cytosol, rendering HMGB1 pro-autophagic. Moreover, HMGB1 translocation is disrupted by suppressing autophagy, suggesting that proper autophagy is required for HMGB1 translocation [126]. HMGB1^−/^^−^ MEF cells showed abolished autophagy stimulation and failed to dissociate Beclin 1 from Bcl-2 during various stimuli, identifying HMGB1 as an important pro-autophagic factor. HMGB1 co-immunoprecipitated with both Bcl-2 and Beclin 1, of which the latter interaction happened indirectly via Bcl-2. This suggests that HMGB1 can regulate autophagy through action on this Bcl-2/Beclin 1 binding (Figure 2). Interestingly, HMGB1 can become oxidized at three cysteines. Tang *et al.* [126] showed that C23 and C45, which form a disulfide bridge upon mild oxidation [131], were important for HMGB1’s interaction with Beclin 1. This protein therefore may represent an important signaling molecule upon reactive oxygen species (ROS) increase, a well-established autophagy inducer [132]. Also, p53 regulates HMGB1-dependent autophagy. p53 can complex with HMGB1 and loss of p53 results in more HMGB1/Beclin 1 association and subsequently increased autophagy. Loss of HMGB1 conversely decreases autophagy. This complex may therefore serve as another important crosstalk point in p53-dependent survival versus death [133]. Next to ROS and p53, the ULK1/Atg13/FIP200 complex, an essential complex for autophagy activation [134], has also been observed as an upstream regulator of the HMGB1/Beclin 1 interaction and thus autophagy. In this respect, the ULK1 complex was requisite for HMGB1 and Beclin 1 to interact [135]. In addition, HMGB1 positively regulates the expression of JNK and ERK, suggesting a secondary indirect mechanism through which HMGB1 can regulate the Bcl-2/Beclin 1 interaction [136]. 

### 5.4. ARF

ARF (p14^ARF^ in humans, p19^ARF^ in mice) is a tumor suppressor, of which a small molecular weight variant was discovered to induce autophagy [137], as well as its full-length variant [138]. This was confirmed by Pimkina *et al.* [139], elucidating a mechanism by which ARF was able to bind to Bcl-X_L_, thereby displacing Beclin 1. Interestingly, p53 knock down increased ARF-induced autophagy, suggesting that also p53 can indirectly affect the Bcl-2/Beclin 1 interaction, in order to regulate autophagy [140]. Importantly, ARF predominantly localizes in the nucleolus and the mitochondria, and overexpression of mitochondria-targeted ARF already had autophagy-promoting effects [139], which is not in line with the findings that only ER-targeted Bcl-2 and Bcl-X_L_ inhibit autophagy. The reason for this is unclear, but close contact sites between mitochondria and ER may allow mitochondrial proteins to affect ER proteins [141].

### 5.5. TRAF6

Stimulation of Toll-like receptors (TLRs) by microbial components like lipopolysaccharides (LPS) leads to recruitment of several typical proteins at the Toll-interleukin-1 receptor (TIR) domain, like myeloid differentiation marker 88 (MyD88), IL-1 receptor-associated kinase (IRAK) 1 and 4, which recruit and activate the K63-linked E3 ubiquitin ligase tumor necrosis factor receptor (TNFR)-associated factor 6 (TRAF6) [142]. Interestingly, Beclin 1 was found to be ubiquitinated after stimulation with LPS. This ubiquitination was K63-linked and dependent on an interaction with TRAF6 [143]. The activation of TRAF6 results, via several other proteins, in the translocation of NF-κB. This transcription factor also induces the production of A20, a deubiquitinating enzyme that terminates the ubiquitinations of TRAF6 [144]. In this respect, A20 was able to reduce the ubiquitination of Beclin 1. The site of ubiquitination was identified as K117, present in the BH3 domain, since mutant K117R Beclin 1 showed less ubiquitination, less autophagy stimulation, less self-association and less PtdIns-3KC3 activity [143]. However, the effect on Bcl-2/Beclin 1 binding was not reported. It is likely though, considering that a former study reported that stimulation of TLR4 also recruited Beclin 1 to this complex, causing dissociation of Beclin 1 from Bcl-2 in macrophages [145] (Figure 2). Intriguingly, this complex forms at the plasma membrane, not at the ER, where Bcl-2 may not be included. However, close-contact sites between plasma membrane and ER exist [146,147]. Otherwise, this complex may be required for the formation of pre-autophagosomal structures deriving from the plasma membrane [10].

### 5.6. Caspases

The cell death proteases, caspases, are important mediators of apoptosis. Recent studies also provided important roles for these proteases in inhibition of autophagy through cleavage of Atg proteins, making them interesting mediators in the crosstalk between these processes [148]. Among these Atg proteins, Beclin 1 can become cleaved by caspase 3 [149,150,151,152,153,154], caspase 6 [149,152], caspase 7 [151], caspase 8 [151,154], caspase 9 and 10 [149]. Cleavage of Beclin 1 can occur at two sites (D133 and D149) [151,154], although D124 has also been proposed [153]. This cleavage results in an inactivation of autophagy. Interestingly, a resulting C-terminal fragment of approximately 35 kDa gained pro-apoptotic functions through its relocalization to the mitochondria [151]. In this manner, when caspases are activated, Beclin 1 can switch from a pro-autophagic to a pro-apoptotic protein. Caspase-cleavage mutants of Beclin 1 therefore sustain the autophagic response and delay apoptosis [154]. This cleavage fragment of Beclin 1 has also been detected in apoptotic cells [151] and astrocytes within neuronal plaque regions [150], suggesting an important physiological role for this cleavage in cellular health and disease.

Zhu *et al.* found that this cleavage also abrogated the Bcl-2/Beclin 1 interaction [153]. However, unlike the other proteins discussed in this review, this caspase-mediated inhibition of the Bcl-2/Beclin 1 binding does not result in increased autophagy, because of the additional inactivation of Beclin 1. The activation of caspases normally occurs after an activation of autophagy, implying that Beclin 1 already became dissociated from Bcl-2. In this respect, the caspase-mediated cleavage of Beclin 1 may simply prevent Beclin 1 from re-associating with Bcl-2, and autophagy to become regulated again. However, caspase inhibitor zVAD prevented caspase-mediated abrogation of the Bcl-2/Beclin 1 interaction, suggesting Beclin 1 can also be cleaved when bound to Bcl-2 [153].

### 5.7. BH3-Only Proteins

Since Beclin 1 binds with its BH3 domain to the hydrophobic groove in Bcl-2, competitors for this binding groove could likely displace Beclin 1 from Bcl-2 (Figure 2). These competitors include the BH3-only proteins and BH3-mimetic molecules, structurally resembling a BH3 domain (see below), but not the multidomain pro-apoptotic proteins Bax and Bak [24]. In this manner, pro-apoptotic proteins also can gain a pro-autophagic role. This was, for example, found for the sole BH3 protein in *C. elegans*, EGL-1. Deletion of EGL-1 blunted starvation-induced autophagy, while a gain-of-function mutation markedly increased autophagy [25].

Bcl-2-associated death promotor (Bad) is an atypical member of the pro-apoptotic Bcl-2 family of proteins, since it does not contain a C-terminal transmembrane domain required for mitochondrial targeting. Maiuri *et al.* [25] found that upon starvation, while Beclin 1 dissociated from Bcl-2 and Bcl-X_L_, the interaction of Bad with those anti-apoptotic proteins increased. The starvation-induced Beclin 1 dissociation and subsequent stimulation of autophagy was attenuated in Bad^−/^^−^ MEF cells, but not autophagy stimulated by ABT-737, suggesting that Bad induced autophagy by interacting with Bcl-2 and hence dissociating Beclin 1 [24]. Also tBid inhibits Bcl-2/Beclin 1 interaction, although the relevance for autophagy has not yet been investigated [24].

Bcl-2/adenovirus E1B 19-kDa-interacting protein 3 (BNIP3) is a BH3-only protein, containing a mitochondria-targeting C-terminal transmembrane domain, which is essential for its pro-apoptotic function [155]. Furthermore, BNIP3 and BNIP3L, a functional homolog of BNIP3, were also found to induce autophagy [156,157,158]. Interestingly, these proteins are well-known targets of the hypoxia-inducible factor HIF1 [159], whose activation upon hypoxic conditions also induces autophagy, more specifically mitophagy, suggesting BNIP3 could be an important factor in this process. Indeed, BNIP3 was required for hypoxia-induced autophagy [156]. Interestingly, hypoxia increased the amount of BNIP3 co-immunoprecipitating with Bcl-2, while decreasing the amounts of Beclin 1, suggesting that BNIP3 competed with Beclin 1 for Bcl-2 binding during hypoxia [156]. However, since BNIP3 is targeted to the mitochondria and only ER-Bcl-2 regulates autophagy, it would be interesting to verify the importance of BNIP3’s C-terminal transmembrane domain during hypoxia-induced autophagy. In this respect, Bellot *et al.* [157] showed that 20-mer BH3 peptides of BNIP3 and BNIP3L were sufficient for stimulating autophagy, even in normoxic conditions, suggesting that BNIP3 C-terminal domain is not required *per se*. BNIP3 was also upregulated and required for autophagy induced by the H-Ras^Val12^ oncogenic mutation, possibly through stimulation of Beclin 1 dissociation from Bcl-2 [160]. Both Atg5 and BNIP3 knockdown led to larger tumors, implying that BNIP3-mediated autophagy is an important tumor suppressor mechanism in Ras-linked tumors. In addition, mutant Ras was found together with overexpression of BNIP3 and increased autophagy levels in the tumors of bladder cancer patients [160], suggesting that modulation of BNIP3-linked autophagy may be of therapeutic interest.

However, this mechanism is not the only manner in which oncogenic Ras promotes autophagy. Ras can also upregulate Atg5 and activate the Rac1/MKK7/JNK pathway [161] and recently, it has been shown to also increase the expression of Beclin 1 and the BH3 protein Noxa [162]. H-Ras^Val12^ induced more Beclin 1 displacement from Mcl-1 and Bcl-X_L_, which was abolished by knock down of Noxa. Here, the effect on Mcl-1 was stronger than that of Bcl-X_L_, probably reflecting the preferential binding of Noxa to Mcl-1. Interestingly, knock down of other BH3 proteins (Bad, Bim and Bmf) had no effect on Ras-induced autophagy, except for Puma, which had a small but significant effect. Also Puma knock down partially abrogated the Ras-induced Beclin 1 dissociation from Mcl-1, albeit not as strongly as Noxa. However, the dissociation from Bcl-X_L_ was more strongly affected with Puma knock down than with Noxa knock down, suggesting that Puma preferentially binds Bcl-X_L_ [162]. Although not fully explored, this report is the first to suggest Puma can also regulate autophagy by altering Bcl-2/Beclin 1 binding. 

Upregulation of Noxa was also involved in autophagy-associated cell death in cancer cells upon co-administration of the ErbB1/2 inhibitor lapatinib and the Bcl-2 family antagonist obatoclax [163]. Here, overexpression of Mcl-1 was most effective in suppressing the induction of autophagy. The synergistic effect of lapatinib and obatoclax diminished Mcl-1/Beclin 1 or Bcl-X_L_/Beclin 1 binding, and increased Mcl-1/Noxa binding, which was counteracted by knock down of Noxa [163]. It is puzzling however that obatoclax, a BH3 mimetic inhibiting Bcl-2, Bcl-X_L_ and Mcl-1 [164], did not affect the Mcl-1/Beclin 1 binding on its own. However, upon co-administration with lapatinib, it was fully dependent on Noxa, which even interacted more strongly with Mcl-1. This suggests that in these conditions, obatoclax did not directly inhibit the Bcl-2 proteins, but acted in another, unknown manner.

### 5.8. Viral Proteins

Infection of cells by viruses will activate autophagy as part of the innate immunity against all kinds of pathogens [165]. However, viruses have developed several protective strategies to affect the autophagic pathway [166], in order to halt the process [167,168] or take it to their advantage to enhance their replication [169] or release the new progeny viruses [170]. Autophagy after infection can be affected thanks to viral proteins, of which some act on the Bcl-2/Beclin 1 interaction.

In this respect, so-called “viral Bcl-2 homologues” have been shown to exist, that can bind Beclin 1, thereby altering the autophagic pathway (Figure 2). This results in an inhibition of Beclin 1, as for the herpes simplex virus protein ICP34.5 [171], the human cytomegalovirus protein TRS1 [172] and γ-herpes viruses viral Bcl-2 homologues, like the Kaposi’s sarcoma-associated herpes virus KSHV Bcl-2 [26] and the γHV68 protein M11 [168,173]. However, binding to Beclin 1 can also enhance the formation of the PtdIns3-KC3 and enhancement of autophagy, as for the adenoviral protein E1B19K [174], which employs the autophagic pathway to release its progeny.

Beclin 1 may also be regulated by viral proteins, in order to modulate other steps in the autophagic pathway, downstream of the Bcl-2/Beclin 1 interaction. The PtdIns3-KC3 namely also affects the fusion mechanisms of autophagosomes with lysosomes, depending on the Beclin1 interactors present in the complex: Atg14L/Barkor, Rubicon or UVRAG [175,176]. Inhibition of Beclin 1 can therefore also affect this function, leading to accumulation of autophagosomes, which has been shown for the human immunodeficiency virus HIV protein Nef [177] and proposed for the influenza A virus matrix protein 2 (M2) [178]. The autophagosomes can be colonized by the viruses as protective environments for virus replication. However, whether these Beclin 1-binding proteins also alter the Bcl-2/Beclin 1 interaction remains elusive.

## 6. Affecting the Bcl-2/Beclin 1 Interaction as Therapy

Since the Bcl-2/Beclin 1 protein complex is a key regulator for autophagy, its modulation through various ways will stimulate or inhibit this cellular pathway (see above). Since modulation of autophagy is proposed as a novel therapeutic avenue for various diseases, including cancer [179], atherosclerosis [180], diabetes mellitus [181], cardiovascular diseases [182] and neurodegeneration [183], affecting this interaction by chemical compounds will likely be of therapeutic relevance. The BH3 domains of these pro-apoptotic proteins are interesting structural templates for the design of BH3-mimetic molecules, able to inhibit the anti-apoptotic functions of Bcl-2 family proteins in cancer cells, thereby triggering cancer cell death [184,185,186]. Although BH3 mimetics were originally designed for induction of apoptosis, their pro-autophagic potential has recently emerged as they can displace Beclin 1 from Bcl-2 proteins, resulting in increased Beclin 1-dependent autophagy. Such effects have been observed with ABT-737 [25,87], (−)-gossypol [187] and Bcl-X_L_ inhibitor Z36, which all bind in the hydrophobic groove [188]. Remarkably, Z18, an analog of Z36 but with lower affinity for Bcl-X_L_, was also shown to induce autophagy (although associated with cell death), but in a Beclin 1-independent manner [30], suggesting another mechanism for this compound, but still involving Bcl-X_L_, since its overexpression could reduce the Z18-induced autophagy. It should be noted however that the dissociation of Beclin 1 is not the only event required for autophagy induction. Indeed, ABT-737 also affected several other autophagy regulators (e.g., AMP-activated kinase AMPK, IκB kinase IKK and mTOR) [189], which were essential for the induction/progress of ABT-737-induced autophagy. However, whether these effects were downstream consequences of the Beclin 1 dissociation was not investigated. The activation of AMPK, IKK and inhibition of mTOR could represent a positive feedback loop upon autophagy initiation. In this respect, knock down of Atg5 or Beclin 1 abolished the ABT-737-induced hyperphosphorylation of IKK and the inhibition of mTOR [189]. A possible mechanism to evoke such feedback regulation may be via the recruitment of Beclin 1 to IP_3_Rs after Beclin 1 dissociation from Bcl-2, thereby provoking an autophagy-stimulating Ca^2+^ release through sensitized IP_3_R channels [104].

Of course, inhibiting the interaction of Beclin 1 with the Bcl-2-family proteins using BH3 mimetics may also trigger apoptosis by releasing pro-apoptotic Bcl-2-family members. To avoid this, low doses of compounds are recommended that mainly affect the weaker Bcl-2/Beclin 1 binding, but not the stronger interactions with pro-apoptotic proteins. In addition, the natural BH3 mimetic (−)-gossypol has been shown to kill cancer cells via autophagic cell death in Bcl-2-proteins-overexpressing, apoptosis-resistant cancer cells [187], strongly suggesting BH3 mimetics’ anti-tumor effects are not solely dependent on apoptosis. However, in other cases, an inhibition of autophagy can be preferential, e.g., in cancer cells that take advantage of autophagy for resistance against nutrient limitations or therapies [179]. In these situations, the enhancement of the Bcl-2/Beclin 1 interaction may be beneficial. Therefore, it is of great importance to further understand the regulation of this interaction and all the players involved, in order to search and design other autophagy-modulating molecules that can have great therapeutic value.

## 7. Conclusions

The Bcl-2/Beclin 1 interaction is a very important checkpoint in the regulation of autophagy and can therefore be regulated by a number of molecules. In regard of the therapeutic possibilities of autophagy modulation, it is crucial to further and fully understand Bcl-2/Beclin 1 regulation. Although a lot of advances have recently been made, many intriguing and important questions regarding this interaction still remain to be investigated.
